# Structural Construction of WO_3_ Nanorods as Anode Materials for Lithium-Ion Batteries to Improve Their Electrochemical Performance

**DOI:** 10.3390/nano13040776

**Published:** 2023-02-20

**Authors:** Yunpeng Zhang, Keke Zhu, Rui Li, Suyuan Zeng, Lei Wang

**Affiliations:** School of Chemistry and Chemical Engineering, Liaocheng University, Liaocheng 252059, China

**Keywords:** WO_3_ nanorod, cyclic stability, lithium-ion batteries

## Abstract

WO_3_ nanobundles and nanorods were prepared using a facile hydrothermal method. The X-ray diffraction pattern confirms that the obtained samples are pure hexagonal WO_3_. Transmission electron microscope images detected the gap between the different nanowires that made up the nanobundles and nanorods. As the anode materials of lithium-ion batteries, the formed WO_3_ nanobundles and WO_3_ nanorods deliver an initial discharge capacity of 883.5 and 971.6 mA h g^−1^, respectively. Both WO_3_ nanostructures deliver excellent capacity retention upon extended cycling. At a current density of 500 mA g^−1^, the reversible capacities of WO_3_ nanobundle and WO_3_ nanorod electrodes are 444.0 and 472.3 mA h g^−1^, respectively, after 60 cycles.

## 1. Introduction

The ever-growing demand for electric vehicles and large-scale grid storage, combined with the limited capacity and rate capability of commercial graphite in lithium-ion batteries (LIBs), has stimulated widespread research into high-performance energy storage electrode materials. Transition metal oxides (TMOs) have become a hotspot as anode material candidates for LIBs in view of their high theoretical capacity, widespread availability, environmental friendliness, and better safety features.

Due to its excellent physiochemical properties, tungsten trioxide (WO_3_) is one of the transition metal oxides that has aroused great attention and presents extensive potential applications in electronics and nanodevices [1,2]. Substantial advancement has been achieved in the energy conversion/storage of WO_3_ nanoparticles. Hexagonal WO_3_ possesses an open-tunnel structure and intercalation chemistry, which contribute to Li^+^ diffusion in the host lattice. However, WO_3_ as an anode in LIBs still suffers from poor capacity retention and rate performance due to its low conductivity and significant volume change during the cycling process [2]. Therefore, the nanostructure of WO_3_ was optimized and modified using intelligent design [3]. It is necessary to use a specially developed theory and ab initio method to explore the formation and stability mechanism of WO_3_ nanorods [4,5]. Meanwhile, the design and construction of hexagonal WO_3_ structures that can accelerate the diffusion of Li^+^ are desirable and interesting.

V.B. Patil et al. synthesized one-dimensional single crystalline tungsten oxide nanorods using a hydrothermal technique. The controlled morphology of tungsten oxide was obtained by using sodium tungstate and oxalic acid as organic inducers [6]. Duan et al. reported a h-WO_3_ biconical mesocrystal that can deliver a discharge capacity of 426 mA h g^−1^ at 50 mA g^−1^ [7]. Huang et al. showed that the initial discharge capacity of hexagonal WO_3_ nanorods was 215 mA h g^−1^ [8]. Qiu et al. found that the porous WO_3_ nanoplate assembly can reversibly deliver a capacity of about 470 mA h g^−1^ at 50 mA g^−1^ [9]. Liu et al. revealed that the discharge capacities of WO_3_ microflowers and WO_3_ nanowires are 549.8 and 503.9 mA h g^−1^ at 200 mA g^−1^, respectively [10]. Many researchers have been involved in developing numerous novel hexagonal WO_3_ nanostructures that can improve lithium storage properties.

In this work, a hydrothermal route was developed for the formation of WO_3_ nanostructures. The lithium storage performance of two formed WO_3_ nanostructures as LIB anodes was investigated and analyzed. The cyclic stability and rate characteristics of the WO_3_ nanorods were superior to those of the nanobundles, which suggested that shape and structure control could exert great influence on the electrochemical properties of the prepared WO_3_ samples.

## 2. Materials and Methods

### 2.1. Synthesis of WO_3_ Nanorods

For the preparation of WO_3_ nanobundles, 1.4432 g Na_2_WO_4_ 2H_2_O was mixed with 30 mL 0.25 mol L^−1^ H_2_SO_4_ under constant stirring. The produced solution was heated at 180 °C for 10 h in an autoclave. The precipitate was centrifuged, washed with water, and dried for characterization and electrochemical measurements. When the initial reaction solution was comprised of 1.4432 g Na_2_WO_4_ 2H_2_O, 20 mL 0.375 mol L^−1^ H_2_SO_4_ and 10 mL glycerol, long WO_3_ nanorods were prepared under the same heating condition.

### 2.2. Morphology and Structure Characterization

The morphology of the samples produced was acquired with a scanning electron microscope (FESEM, Thermo Fischer, Helios CX, Waltham, MA, USA). Microstructures and elemental mapping images were detected using a high-resolution transmission electron microscope (HRTEM, Thermo Fischer, Talos F200x, Waltham, MA, USA). X-ray powder diffraction (XRD) patterns of the samples were recorded on a diffractometer (Rigaku Smartlab 9, Tokyo, Japan) with Cu Kα radiation (λ = 1.5406 Å) during a scan range of 10–80° at a scan rate of 20°/min. X-ray photoelectron spectroscopy (XPS) was determined through an X-ray photoelectron spectrometer (ESCALAB Xi+). Fourier transform infrared (FT-IR) spectra were measured with an infrared spectrometer (Nicolet 6700). Thermogravimetric analysis was conducted using a thermal gravimetric analyzer (NETZSCH STA F5, Selb, Germany) with a heating rate of 10 °C min^−1^. The BET surface areas as well as size distributions for the samples were measured using an N_2_ adsorption–desorption instrument (Micromeritics ASAP 2460, Norcross, GA, USA).

### 2.3. Electrochemical Studies

In order to study the lithium storage property of WO_3_ nanostructures, WO_3_ anodes were prepared first. WO_3_ samples were blended mechanically with acetylene black and PVDF at a mass ratio of 70:20:10; the created slurry was pasted onto Cu foil, dried at 80 °C under vacuum for 12 h. The copper foil was then cut into 1.2 cm diameter discs and utilized as the working electrode. Lithium foil acted as the counter electrode. The electrolyte utilized in this work was 1.0 mol L^−1^ LiPF_6_ (EC/DEC, 1:1 in volume, 1 mol L^−1^) with fluoro-ethylene carbonate (FEC, 5% in weight) additive agent. A Celgard 2400 polypropylene membrane served as the separator. The assembly process of the CR-2032 coin-type cells was performed in a glove box filled with argon.

Galvanostatic charge–discharge (GCD) tests were conducted on CT2001A LAND (Wuhan, China) battery testing systems in a voltage range of 0.1−3.0 V (vs Li^+^/Li) at a current density of 0.5 A g^−1^. The electrochemical tests were implemented on a ZAHNER Zennium Pro electrochemical workstation. Cyclic voltammetry (CV) was determined in a voltage range of 0.01−3.0 V (vs. Li^+^/Li) at a scanning rate of 0.1 mV s^−1^, while electrochemical impedance spectroscopy (EIS) was measured within the frequency bandwidth of 0.01–10^5^ Hz at an amplitude of 10 mV.

## 3. Results and Discussion

### 3.1. Composition and Microstructures of WO_3_

**Figure 1:**
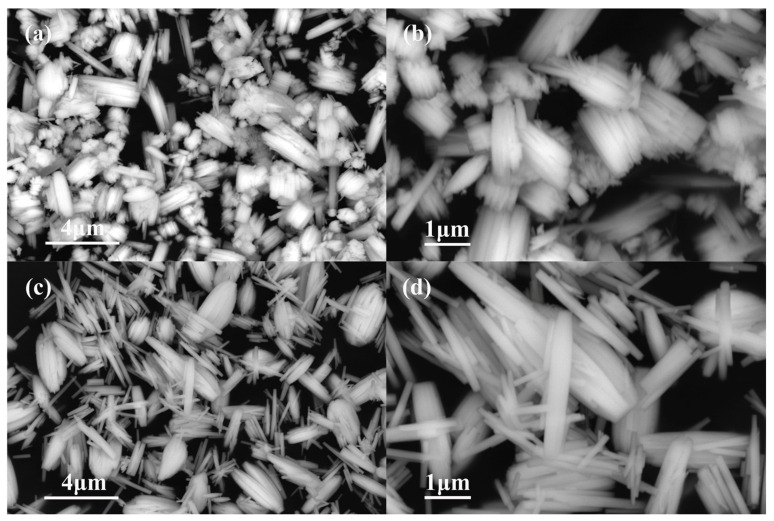
FE-SEM images of the products formed with different initial reactants. (**a**,**b**) 1.4432 g Na_2_WO_4_ 2H_2_O, 30 mL 0.25 mol L^−1^ H_2_SO_4_ (**c**,**d**) 1.4432 g Na_2_WO_4_ 2H_2_O, 20 mL 0.375 mol L^−1^ H_2_SO_4_, and 10 mL glycerol.

Generally, the size of WO_3_ nanorods is in the range of 30 nm–5 μm [6,11]. The shape, morphology, and size of the two samples we prepared can be denoted by the FESEM image. For the product prepared from 1.4432 g Na_2_WO_4_ 2H_2_O and 30 mL 0.25 mol L^−1^ H_2_SO_4_, the image depicts the formation of nanobundles of different sizes (Figure 1a). The higher-magnification image shows that the bundles are composed of nanorods with a length of 1–3 μm and diameter of 0.2–1 μm (Figure 1b). As the initial reactants were changed to 1.4432 g Na_2_WO_4_ 2H_2_O, 20 mL 0.375 mol L^−1^ H_2_SO_4_, and 10 mL glycerol, the formed product was mainly composed of nanorods (Figure 1c) about 2 μm in length and 0.2 μm in diameter (Figure 1d). The results indicated that the addition of glycerol may increase the viscosity of the initial reaction solution, influencing the aggregation and the ratio of the length to the diameter of the nanorods, which facilitated the formation of more uniform nanorods.

**Figure 2:**
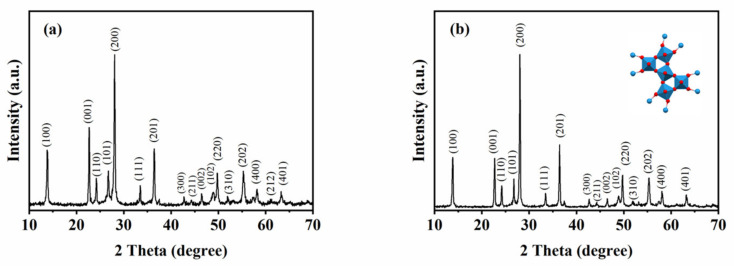
XRD patterns of the formed products with different initial reactants. (**a**) 1.4432 g Na_2_WO_4_ 2H_2_O, 30 mL 0.25 mol L^−1^ H_2_SO_4_ (**b**) 1.4432 g Na_2_WO_4_ 2H_2_O, 20 mL 0.375 mol L^−1^ H_2_SO_4_, and 10 mL glycerol.

The atomic structure of the samples was characterized and analyzed (Figure 2). XRD patterns denote the family of crystal planes (100), (001), (110), (101), (200), (111), (201), (300), (211), (002), (102), (220), (310), (202), (400), (212), and (401) at the 2 theta angles 13.96°, 22.76°, 24.29°, 26.87°, 28.16°, 33.54°, 36.47°, 42.72°, 44.31°, 46.52°, 48.92°, 49.78°, 52.13°, 55.33°, 58.25°, 61.23°, and 63.42°. The peaks and crystallographic planes detected above were interrelated in the hexagonal phase of the WO_3_ (JCPDS card no. 75-2187), which demonstrated that the two samples are h-WO_3_. The inset in Figure 2b shows the crystal structure of h-WO_3_.

**Figure 3:**
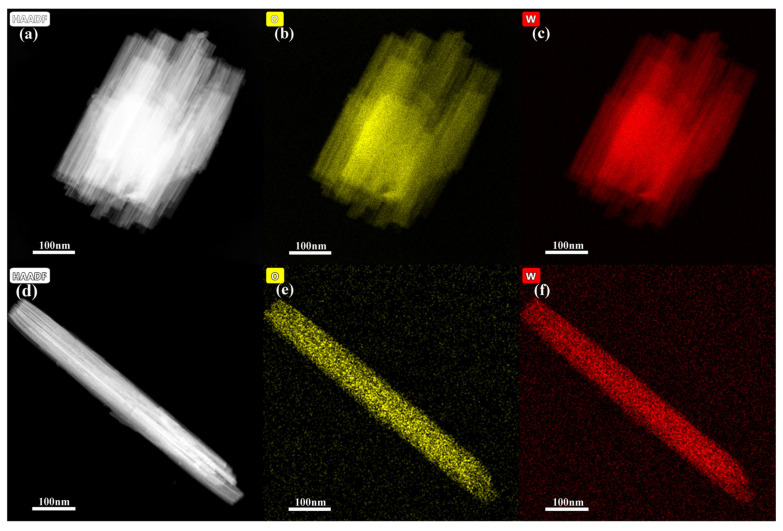
Elemental mapping images of the formed products with different initial reactants. (**a**–**c**) 1.4432 g Na_2_WO_4_ 2H_2_O, 30 mL 0.25 mol L^−1^ H_2_SO_4_, (**d**–**f**) 1.4432 g Na_2_WO_4_ 2H_2_O, 20 mL 0.375 mol L^−1^ H_2_SO_4_, and 10 mL glycerol.

EDAX elemental mapping was investigated to confirm the elemental composition of the formed WO_3_ samples (Figure 3). The presence of W and O with uniform distribution further demonstrates that the formed products are WO_3_. HAADF images demonstrated that both the formed nanobundles and nanorods are composed of many nanowires, which also proves that the composition of initial reactants influences the shape and structure of the product.

**Figure 4:**
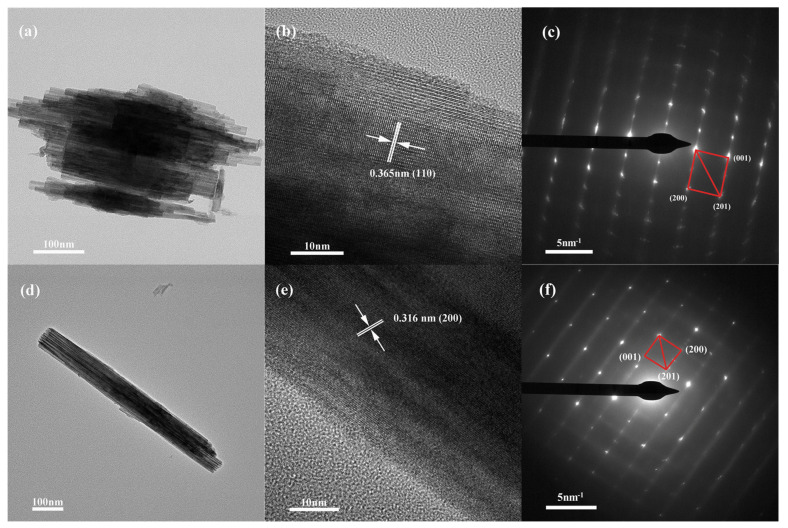
TEM, HR-TEM, and SAED images of the products created with different initial reactants. (**a**–**c**) 1.4432 g Na_2_WO_4_ 2H_2_O, 30 mL 0.25 mol L^−1^ H_2_SO_4_, (**d**–**f**) 1.4432 g Na_2_WO_4_ 2H_2_O, 20 mL 0.375 mol L^−1^ H_2_SO_4_, and 10 mL glycerol.

TEM, HRTEM, and SAED images of the two h-WO_3_ samples were analyzed (Figure 4). For the h-WO_3_ prepared with 1.4432 g Na_2_WO_4_ 2H_2_O and 30 mL 0.25 mol L^−1^ H_2_SO_4_, nanorods can be clearly observed in the bundle-like particle (Figure 4a). The h-WO_3_ nanorods are composed of nanowires with a diameter of 10 nm and a length of around 150–450 nm. HRTEM imaging proves that a spacing of 3.65 Å can be assigned to the (110) plane of h-WO_3_ (Figure 4b). Figure 4c shows the selected electron diffraction pattern of h-WO_3_ nanobundles. The diffraction spots are in general agreement with the crystal planes (or their equivalent planes) (001), (200), and (201). For the h-WO_3_ prepared using 1.4432 g Na_2_WO_4_ 2H_2_O, 20 mL 0.375 mol L^−1^ H_2_SO_4_, and 10 mL glycerol, nanorods with a length of 700 nm and diameter of 80 nm can be seen (Figure 4d). The nanorod is constructed with various nanowires that are about 10 nm in diameter and 700 nm in length. The lattice fringes are perpendicular to the nanorod axis and the distance of 3.16 Å coincides with the (200) crystal plane of h-WO_3_ (Figure 4e). Figure 4f shows the selected electron diffraction pattern of h-WO_3_ nanorods. Selected area electron diffraction pattern (SAED) indicates that the h-WO_3_ nanorod is a single crystal. The diffraction spots are in general agreement with the crystal planes (or their equivalent planes) (001), (200), and (201). The results are consistent with those of XRD and HRTEM above.

**Figure 5:**
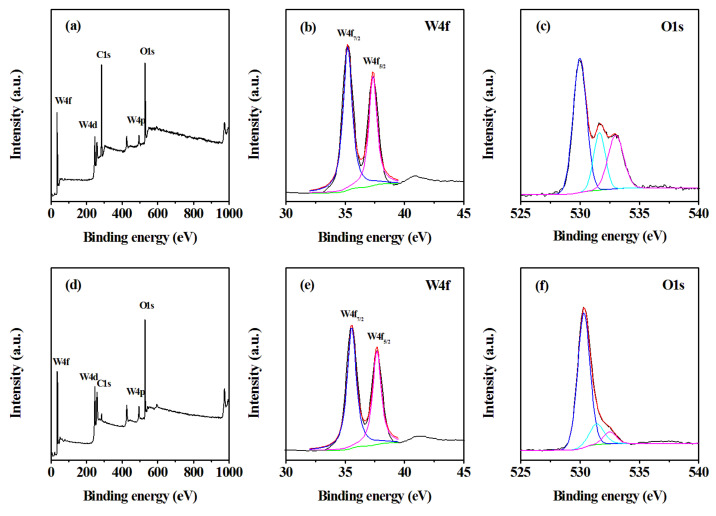
XPS spectra of the formed WO_3_ with different shapes. (**a**–**c**) WO_3_ nanobundles, (**d**–**f**) WO_3_ nanorods.

XPS was utilized to study the chemical states of as-prepared samples (Figure 5). The full wide-scan spectrum of the sample h-WO_3_ nanobundles and nanorods are presented in Figure 5a,d respectively. The characteristic peaks of elements W and O could be clearly observed. For W 4f XPS spectra of the formed h-WO_3_ nanostructures, the two peaks that appeared at 35.4 and 37.6 eV are mainly from W 4f7/2 and W 4f5/2 of h-WO_3_, respectively, which demonstrates that the oxidization states of W atoms in the two nanostructures are +6 [12,13]. The O 1s XPS spectra of two h-WO_3_ nanostructures present obvious differences. Three fitting peaks at 530.2, 531.6, and 532.7 eV derive from the crystal lattice oxygen O^2−^ of WO_3_, O^2−^ ions in oxygen-deficient regions within the matrix of WO_3_, and the adsorbed H_2_O on the surface of WO_3_, respectively [14,15]. For the O 1s XPS of WO_3_ nanobundles, quantitative analysis suggested that the normalized oxygen specimen percentages in the W-O bond, oxygen components in the oxygen vacancy, and the chemisorbed and dissociated oxygen species are 54.35%, 20.29%, and 25.36%, respectively. For the O 1s XPS spectrum of WO_3_ nanorods, we also used the integral area normalization method to obtain the normalized contents of the above three oxygen species of 78.03%, 14.65%, and 7.32%, respectively. The variation of lattice oxygen O^2−^ and adsorbed oxygen components is in line with the EDS quantitative results, which further proves that the initial reactants can change the chemical composition of products.

**Figure 6:**
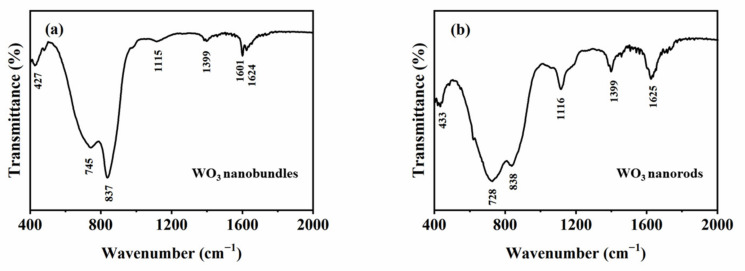
FT-IR spectra of the formed WO_3_ with different shapes. (**a**) WO_3_ nanobundles, (**b**) WO_3_ nanorods.

The FTIR spectra of the WO_3_ samples were detected and analyzed (Figure 6). It has been reported that h-WO_3_ comprises packed corner-sharing WO_6_ octahedra that possess a vibration mode in the region of 1000–600 cm^−1^ [13,16]. The peaks at 745 and 837 cm^−1^ can be thought of as a shortening of W–O bonds [13], while the broad peak at 728 cm^−1^ occurs in the stretching vibration of W–O_inter_–W of bridged corner-sharing WO_6_ octahedra in h-WO_3_ [17,18]. The peak at 1624 cm^−1^ is born out of the δ(OH) modes of adsorbed water molecules; the weak peak that appears at 1399 cm^−1^ is thought to be the unusual values of bending δ(OH) vibrations [13]. FT-IR spectra analysis confirmed that the two products are h-WO_3_.

**Figure 7:**
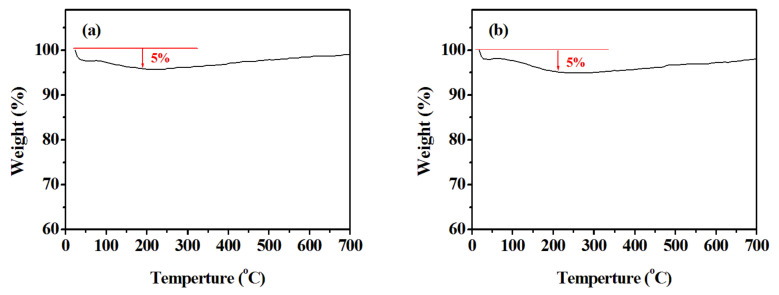
TG analysis of the formed WO_3_ with different shapes. (**a**) WO_3_ nanobundles, (**b**) WO_3_ nanorods.

The weight-loss process of the WO_3_ nanostructures was investigated using TG analysis. As observed in Figure 7, Both WO_3_ nanobundle and nanorod structures have a certain degree of mass loss below 200 °C of about 5%.

**Figure 8:**
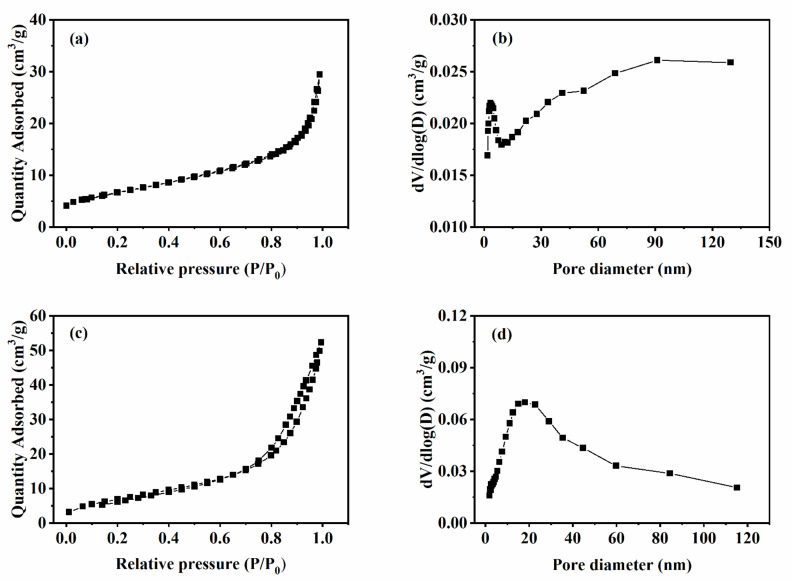
N_2_ adsorption–desorption isotherm and pore size distribution of the formed WO_3_ with different shapes. (**a**,**b**) WO_3_ nanobundles, (**c**,**d**) WO_3_ nanorods.

The N_2_ adsorption–desorption isotherms and size distributions for all samples were also measured (Figure 8). The Brunauer–Emmett–Teller (BET) surface areas of the WO_3_ nanobundles and nanorods were determined to be 23.52 and 26.49 cm^2^/g, respectively. A desorption isotherm was used to determine the pore size distribution via the Barret–Joyner–Halender (BJH) method. Nitrogen adsorption volume at a relative pressure (P/P_0_) of 0.994 was used to determine the pore volume and average pore size. The pore volumes of WO_3_ nanobundles and nanorods were 0.045 and 0.081 cm^3^/g, respectively. The average pore sizes were 8.89 and 10.77 nm. Obviously, after the WO_3_ changed from bundle structure to rod structure, the specific surface area, pore volume, and average pore size of the material was greatly improved.

### 3.2. Electrochemical Performance of WO_3_

**Figure 9:**
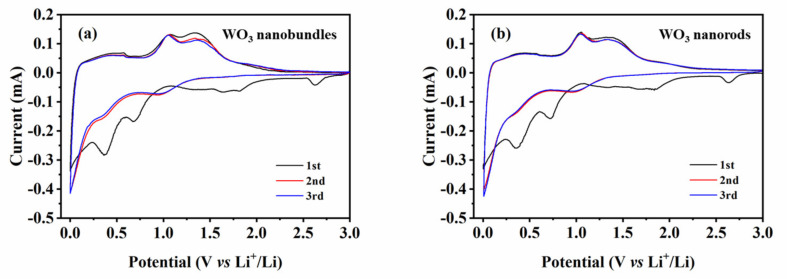
Cyclic voltammograms of WO_3_ nanostructures with different shapes at 500 mA g^−1^ in a voltage range of 0.01–3 V. (**a**) WO_3_ nanobundles, (**b**) WO_3_ nanorods.

Cyclic voltammograms of the WO_3_ nanobundles and nanorods at a scanning rate of 0.1 mV s^−1^ were tested to investigate their electrochemical lithium storage properties (Figure 9). In the initial cycle, four reduction peaks at approximately 2.63, 1.65, 0.69, and 0.37 V can be found. The reduction peak at 2.7 V corresponds to Li^+^ intercalation into WO_3_ [19], the broad weak peak at 1.65 V comes from the lithium insertion process [20] and the kinetic nature of WO_3_ decomposition [21], and the peak at 0.7 V is down to the reductive conversion of WO_3_ to metal W [19], while the reduction peak at 0.37 V occurs in the irreversible insertion of lithium into the WO_3_ crystal lattice [20]. The disappearance of the above reduction peaks suggests that the irreversible capacity loss may be mainly from the unrecoverable phase transformation and the appearance of a solid electrolyte interphase (SEI) [22,23]. It has been reported that at least two processes are involved in Li^+^ intercalation into WO_3_ crystals [10]. The fast Li^+^ insertion process that occurs in a disordered way results in the decrease of interlayer distance, while the slower Li^+^ diffusion and residence process can cause two-dimensional structural relaxation within WO_3_ layers. The lithium insertion process is accompanied by the reduction of the tungsten specimen from a high valence state (W^6+^) to a low valence state (W^0^); considerable changes in crystal structure are induced with massive lithium ion insertion. In the first anodic process, two oxidation peaks at 1.08 and 1.36 V are presented, which originate from the lithium extraction process and the oxidation reaction of W (W^0^ → W^6+^). During the second and third cycles, one broad reduction peak can be identified at 1.0 V, whereas the anodic polarization process is similar to that which occurs in the first cycle, which further demonstrates recoverable phase transformation in the subsequent cycles. The electrochemical lithium storage process for WO_3_ nanostructures can be expressed as the following [10]:

$${\mathrm{WO}}_{3}+6{\mathrm{Li}}^{+}+6e^{-}↔W+3{\mathrm{Li}}_{2}O$$

**Figure 10:**
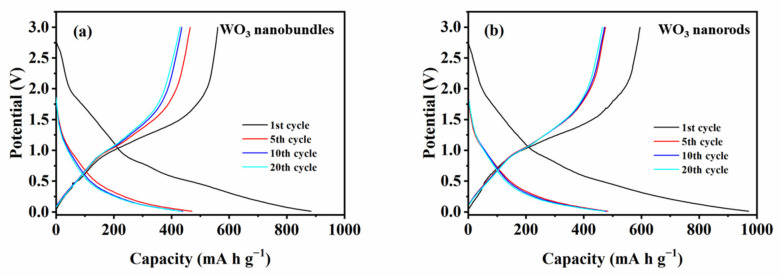
Galvanostatic discharge/charge curves of WO_3_ nanostructures with different shapes at 500 mA g^−1^ in a voltage range of 0.01–3 V. (**a**) WO_3_ nanobundles, (**b**) WO_3_ nanorods.

The GCD curves of WO_3_ nanobundles and nanorods during the 1st, 5th, 10th, and 20th cycles were analyzed at 25 °C (Figure 10). the capacity difference of two WO_3_ samples in the first cycle can be confirmed. The small voltage plateau at 0.85 V mainly originated from electrolyte decomposition and the formation of an SEI film on the Li-WO_3_ [20]. Furthermore, the initial voltage plateau in the subsequent cycles became less obvious, which confirmed that irreversible capacity loss occurred. For the WO_3_ nanobundle electrode, the discharge/charge capacities in the 1st, 5th, 10th, and 20th cycles were 883/560, 478/473, 470/465, and 438/435 mA h g^−1^, respectively. For the WO_3_ nanorods, the tested discharge/charge capacities in the 1st, 5th, 10th, and 20th cycles were 971/595, 483/471, 478/471, and 471/464 mA h g^−1^, respectively. GCD testing results confirm the high initial lithium storage capacity and capacity retention property of WO_3_ nanobundles and WO_3_ nanorods.

**Figure 11:**
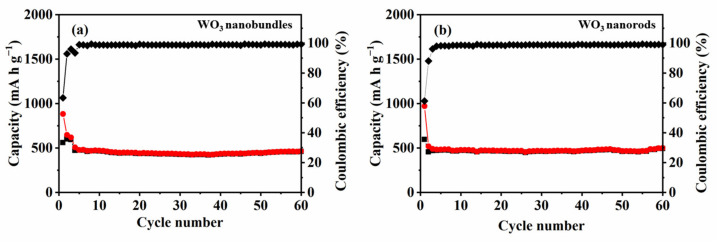
Cycle performances of WO_3_ nanostructures with different shapes at 500 mA g^−1^ in a voltage range of 0.01–3 V. (**a**) WO_3_ nanobundles, (**b**) WO_3_ nanorods.

The cycling performances of the two WO_3_ samples were investigated at 25 °C (Figure 11). The initial discharge capacity of WO_3_ nanobundles and nanorods reached 883 and 971 mA h g^−1^, respectively. Subsequently, the reversible capacities of the two samples gradually decreased. Both WO_3_ nanobundle and nanorod electrodes exhibited a considerable initial irreversible capacity loss, which was born out of the reductive transformation from WO_3_ to W, the structural organization of the prepared samples, and the appearance of an SEI film [10,24]. In the second cycle, neither nanobundle nor nanorod electrodes showed a large loss of capacity. It is quite evident that both of the WO_3_ nanostructures possess excellent capacity retention upon extended cycling. After 60 cycles, the discharge capacity of WO_3_ nanobundles and WO_3_ nanorods is 444 and 472 mA h g^−1^, respectively. It was reported that hexagonal WO_3_ nanorods delivered a maximum discharge capacity of 215 mA h g^−1^ at the initial cycle [8] and hexagonal WO_3_ nanowires presented an initial discharge capacity of 218 mA h g^−1^ [25]. WO_3_ hollow nanospheres and dense particles can deliver an initial discharge capacity of 1054 and 713 mA h g^−1^, respectively, while their discharge/charge capacities in the 50th cycle change into 332/327 and 66/66 mA h g^−1^ [26]. Furthermore, the reversible capacity values of WO_3_ nanobundles and nanorods are higher than graphite (372 mA h g^−1^), which establishes that the two prepared WO_3_ nanostructures can be promising anodes for LIBs.

The results also indicate that the calculated coulombic efficiency after four cycles is higher than 98.0%, which suggests that the WO_3_ nanobundle- and nanorod-based electrodes are highly stable during repeated charge–discharge cycles. However, coulombic efficiency increases significantly with an increase in current density. There are two main reasons for this. The first is probably due to shorter charging/discharging time and lower charge loss at high current densities and the morphology of WO_3_ itself [27]. Another reason is that more irreversible reactions will occur in the first three small current charge–discharge activation processes, leading to low coulombic efficiency. After 50 cycles, the WO_3_ nanobundle and nanorod electrodes retained about 50.3% and 48.6% of their initial capacity, respectively. Furthermore, the reversible capacity of nanorod electrodes is larger than that of nanobundles, which demonstrates that the shape of an as-synthesized product can influence the electrochemical lithium storage performance.

**Figure 12:**
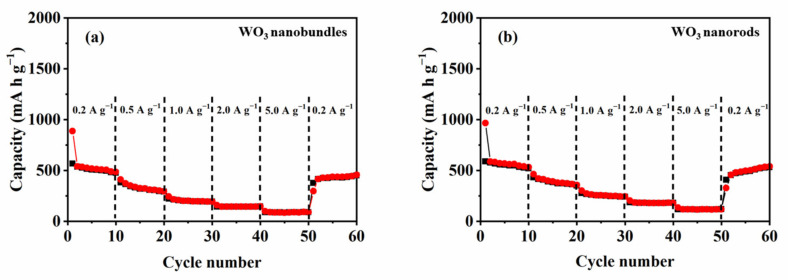
The rate performance of WO_3_ nanostructures with different shapes. (**a**) WO_3_ nanobundles, (**b**) WO_3_ nanorods.

The rate performances of the two WO_3_ nanostructure-based electrodes were investigated and analyzed at 25 °C (Figure 12). The results indicate that the charge/discharge capacity decreases along with increased current density. For the WO_3_ nanobundle electrode, it delivers a discharge capacity of 513, 325, 201, 144, 88, and 436 mA h g^−1^ at a current density of 200, 500, 1000, 2000, 5000, and 200 mA g^−1^, respectively. (Retention: 84.50%; 0.2–0.2 A g^−1^ in the last cycle). For the WO_3_ nanorod electrode, the discharge capacity drops steadily from 561, 386, 254, 181, and 118 mA h g^−1^ as the current density increases from 200, 500, 1000, and 2000 to 5000 mA g^−1^, respectively. When the current density is again lowered to 200 mA g^−1^, the obtained discharge capacity is 504 mA h g^−1^ (retention: 89.8%; 200–200 mA g^−1^ in the last cycle), which is larger than that of reported WO_3_ microflowers and nanowires after a discharge rate of 1600 mA g^−1^ [10]. These results confirm the high structural stability of WO_3_ nanorod and nanobundle electrodes, and WO_3_ nanorods delivered better rate performance than the nanobundles.

**Figure 13:**
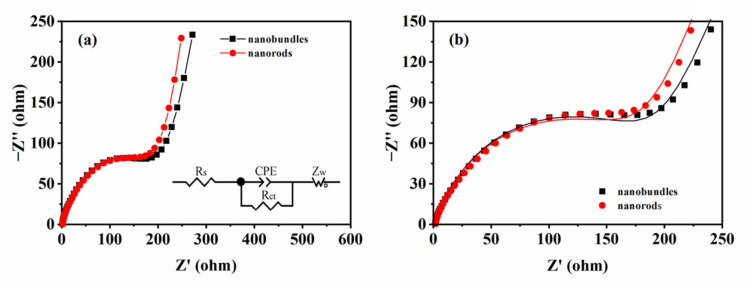
Nyquist plots of WO_3_ nanostructures with different shapes (**a**) and corresponding enlarged fitting plots (**b**). The inset shows the equivalent electrical circuit used.

The electrochemical impedance spectra of the WO_3_ nanobundle and nanorod electrodes were investigated in terms of Nyquist plots and equivalent circuits obtained by data fitting (Figure 13). The results indicated the electrolyte resistance (R_s_) value of both WO_3_ nanobundles and nanorods is about 1.5 Ω, while the charge transfer resistance value obtained from equivalent circuit fitting is around 184 Ω for the nanobundle electrode and 175 Ω for the WO_3_ nanorod electrode. In addition, the larger slope at low frequency indicates fast electrolyte ion transfer in WO_3_ nanorod electrodes.

Such excellent rate performance and reversibility of WO_3_ nanobundles and nanorods could be assigned to their comparable uniform structures and small sizes. The nanowires that constitute the nanobundles and nanorods can effectively reduce Li^+^ diffusion distance, present better accommodation of structural strain, and enhance lithium storage performance. Similar cases have been reported in symmetrical 3D chrysanthemum-like WO_3_·0.33H_2_O, WO_3_ microflowers and nanowires, and hierarchical WO_3_ flowers [9,10,28]. In addition, the small-size effect of WO_3_ nanostructures will be conducive to improving electrochemical reactivity and enabling the reversible conversion reaction between Li^+^ and Li_2_O [10,29,30].

## 4. Conclusions

In summary, a mild hydrothermal condition was utilized for the preparation of WO_3_ nanorods and nanobundles. TEM observation showed that the formed nanorods and nanobundles are constructed with nanowires with a diameter of 10 nm. The formed WO_3_ nanostructure electrodes presented high initial discharge capacities and excellent reversible capacities after 60 cycles. The enhanced electrochemical lithium storage property is mainly from the nanosize effect and the void space between the different nanowires that make up the nanorod, which facilitates fast lithium intercalation/de-intercalation kinetics.

## Data Availability

The data presented in this study are available on request from the corresponding author.
